# From framework to practice: applying the proteus-practice framework to learn lessons for integrating PROs in clinical care

**DOI:** 10.1007/s11136-026-04297-7

**Published:** 2026-06-15

**Authors:** Anne L R Schuster, Claire Snyder, Lotte Haverman, Jennifer R Cracchiolo, Liv Marit Valen Schougaard, Amy M Cizik, Alex Tarlochan Singh Sandhu, Michael Brundage, Norah L Crossnohere

**Affiliations:** 1 Department of Biomedical Informatics, The Ohio State University College of Medicine, Columbus, OH USA; 2 Departments of Medicine, Oncology, and Health Policy & Management, Johns Hopkins Schools of Medicine and Public Health, Baltimore, MD USA; 3 Child and Adolescent Psychiatry & Psychosocial Care, Amsterdam UMC location University of Amsterdam, Emma Children’s Hospital, Amsterdam, The Netherlands; 4 Head and Neck Service, Department of Surgery, Memorial Sloan Kettering Cancer Center, New York, NY USA; 5 AmbuFlex, Centre for Patient-Reported Outcomes, Gødstrup Hospital, Herning, Denmark; 6 Department of Clinical Medicine, Aarhus University, Aarhus, Denmark; 7 Department of Orthopedics, University of Utah, Salt Lake City, UT USA; 8 Department of Medicine, Stanford University School of Medicine, Stanford, CA USA; 9 Queen’s University Sinclair Cancer Research Institute, Cancer Care and Epidemiology, Kingston, ON Canada; 10 Division of General Internal Medicine, Department of Internal Medicine, The Ohio State University College of Medicine, Columbus, OH USA

**Keywords:** Patient-reported outcomes, Clinical practice, Implementation science, Real-world evidence

## Abstract

**Purpose:**

As the use of patient-reported outcomes (PROs) in clinical practice expands, there is a growing need to understand how PROs are being integrated across diverse healthcare settings. We aimed to use the PROTEUS-Practice Framework to systematically describe real-world approaches to PRO integration into clinical care and identify lessons learned.

**Methods:**

Participants in the international PROTEUS Consortium were invited to submit case studies describing PRO applications in clinical care. Contributors completed a structured template aligned with the PROTEUS-Practice Framework, which outlines 16 steps for PRO integration across design, implementation, and management stages. Case studies were descriptively summarized by framework step and responses were thematically analyzed by framework stage to identify lessons learned.

**Results:**

Five case studies described the integration of PROs in diverse clinical contexts, including three within individual health systems and two spanning multiple health systems. Fifteen cross-cutting lessons learned emerged across the PROTEUS-Practice Framework stages. Themes identified in at least three case studies include the importance of stakeholder training during design, PRO champions during implementation, and EHR integration to support management. Clear goals, alignment of expected outcomes, iterative design processes, user-friendly visualizations, and regular system updates were also commonly emphasized as critical to successful integration.

**Conclusion:**

The PROTEUS-Practice Framework provided a structured approach for describing real-world approaches to PRO integration in clinical care. Its use enabled the identification of cross-cutting lessons learned that offer practical guidance for optimizing PRO integration and tailoring its use in diverse clinical contexts.

## Introduction

Patient-reported outcomes (PROs) are direct reports from patients about their health status, symptoms, or quality of life, without interpretation by clinicians or others [1, 2]. PROs are typically captured using standardized and validated PRO measures (PROMs). Supported by growing evidence that PROM use can improve patient care and outcomes [3–7], they are increasingly used in clinical practice.

A growing body of literature examines the barriers and facilitators to implementing PROMs in clinical practice, as well as presents case studies applying general implementation science frameworks to guide PROM integration across diverse healthcare settings [8, 9]. These studies and general implementation science frameworks have provided valuable insights into common challenges—such as technological limitations, workflow disruptions, and uncertainty about the utility of PROMs—as well as context-specific enablers and implementation strategies [10, 11]. However, while general implementation science frameworks are well suited to identifying determinants of adoption, they do not explicitly highlight PRO-specific operational decisions required for effective integration of PROs into clinical care such as goal setting, PRO and measure selection, timing and frequency of PROM collection, presentation and visualization of PRO results, electronic health record (EHR) integration, and governance structures.

The PROTEUS Guide to Implementing Patient-Reported Outcomes in Clinical Practice: A Synthesis of Resources (The PROTEUS-Practice Framework) can complement general implementation science frameworks by offering a comprehensive, specific, and structured approach for integrating PRO systems into clinical care [12]. It was published in 2023 and synthesizes key resources [13–16] to describe 16 steps for PRO integration across the stages of Design, Implementation, and System and Data Management [17]. The framework emphasizes that PRO systems must be tailored to particular healthcare settings, system goals, and patient populations as there is no one-size-fits all approach. There is a need for detailed, practice-based accounts of how PRO systems are being integrated into clinical care that address these considerations comprehensively.

To address this need, we sought to use the PROTEUS-Practice Framework to systematically describe real-world approaches to PRO integration and identify lessons learned from implementing PROs in clinical care. By mapping real-world approaches to the PROTEUS-Practice Framework and identifying cross-cutting lessons learned, this work offers practical guidance for optimizing PRO integration. These insights provide in-depth information that can support tailoring PRO integration in diverse clinical settings.

## Methods

We used a case study approach to explore how and why decisions regarding PRO intervention were made. Case study approaches explore contemporary phenomena in depth and within their real-world context [18]. Such approaches can be particularly valuable when it is difficult to disentangle the phenomena and the context.

Case studies for inclusion were identified by referral from members of the Patient-Reported Outcomes Tools: Engaging Users and Stakeholders (PROTEUS) Consortium. PROTEUS is an international network of more than 65 organizations representing key stakeholder groups involved in the use of PROs, including those with experience in research, clinical trials, universities and health systems, and patient and clinician advocacy groups [19]. PROTEUS leadership invited consortium participants to contribute case studies reflecting a range of applications, including system-wide implementations and disease-specific initiatives, with which the PROTEUS leadership team had not been involved. Because the PROTEUS Consortium includes participants from a broad range of relevant stakeholders, only the subset of individuals affiliated with universities and/or health systems was likely positioned to contribute a case study.

Data collection and analysis of the case studies was guided by a retrospective application of the PROTEUS-Practice Framework for purposes of providing a consistent organizing structure to facilitate case study descriptions and cross-case learning. For each case study, contributors completed a structured template aligned with the PROTEUS-Practice Framework describing how their PRO systems addressed each of the 16 steps (Table 1). As part of the structured template, case study contributors were asked to reflect on and describe key lessons learned across each stage of the PROTEUS-Practice framework, including what worked well, what challenges they encountered, and what recommendations they would make to do things differently. Case studies were descriptively summarized by framework step with the PROTEUS-Practice Framework applied as a retrospective, descriptive organizing structure rather than as an evaluative or comparative tool. Thematic analysis was used to synthesize lessons learned by framework stage to identify cross-cutting lessons learned and unique context-specific insights [20, 21]. To mitigate potential bias, analytic roles were deliberately separated between case study contributors and PROTEUS leadership team analysis. Specifically, members of the PROTEUS leadership team who were not involved in the design, implementation, or management of any individual case study led the summary of each case study and the synthesis of key lessons learned. Case study contributors reviewed the summarized descriptions and syntheses for accuracy.

**Table 1:** PROTEUS-Practice Framework Overview

PROTEUS-Practice Framework: stages-steps	Key points related to each step
*Design stage*	
1. Goals	Whether the PRO system is designed to enhance individual patient care, improve population health, facilitate research, enhance clinical datasets, or multiple of the above
2. Barriers & Facilitators	Barriers and facilitators encountered at the patient-, provider-, administrator-, and system- level
3. Training & Engagement	Relevant perspectives engaged in the design, development, and implementation of the PRO system; training content and modality for the various stakeholders
4. Identifying Patients	Which patients complete PROMs (e.g., all patients in the healthcare system, patients in a specific clinic, patients with a defined condition, patients receiving a specific treatment/intervention)
5. Outcomes & Measures	Outcomes assessed and PROMs used
6. Frequency & Timing	When (e.g., at visits and/or between visits) and how often (e.g., one time, multiple timepoints) PROMs are assessed
*Implementation stage*	
7. Administering & Scoring	Whether PROMs are collected within or outside clinical settings; collection modalities used (e.g., self- or-interviewer administration via paper, telephone, internet); how PROMS are scored (e.g., automatically or manually)
8. Workflow	How PROMs are: (1) deployed to patients for completion; (2) collected from patients; (3) tracked for completion; (4) reviewed by the clinical team; (5) documented in the system
9. Results Presentation	Whether PRO results are presented before, during, and/or after a clinical visit; displayed using numeric or visual modalities; and include reference values to inform interpretation
10. Visualizations	How information to aid interpretation such as score directionality, meaning, and possibly concerning results are indicated visually
11. Responding to Issues	How clinical teams are notified about and expected to respond to possibly concerning scores
*System & Data Management stage*	
12. Evaluation	Whether implementation and/or patient clinical outcomes are assessed to evaluate PROM program impact
13. EHR Integration	Whether and how PRO data are uploaded to the EHR
14. Governance	How the PRO system is governed (e.g., disciplines represented and extent to which governance is centralized)
15. Data Pooling/Exchanging	Whether and how the PRO system data are pooled/exchanged with others for multi-institutional analyses
16. Ethical/Legal Issues	Approaches to disclosure; whether and how inclusion of diverse patient populations is supported

*PRO* patient-reported outcome,

*PROM* patient-reported outcome measure,

*EHR* electronic health record

## Results

Five case studies were received, and all were included. They reflect various healthcare contexts, clinical applications, and geographic settings. Importantly, the included case studies also vary in their timing of PRO integration and maturity, with initial implementations spanning ten years and some predating the availability of formal PRO implementation guidance by more than a decade. Three case studies focus on the integration of PROs within an individual health system in the United States, with two focused on specific clinical applications in oncology and heart failure (Table 2a). Two case studies describe PRO integration efforts spanning multiple health systems (Tables 2b) within two European countries.

**Table 2a.** Case studies of PRO integration in individual health systems, summarized using the PROTEUS-Practice Framework

PROTEUS-Practice Framework step	University of Utah health system	Memorial Sloan Kettering Cancer Center’s surgery recovery tracker	Stanford’s Heart Failure Clinic
1. Defining Goals	Collecting PROs clinically since mid-2013 as part of an initiative known as mEVAL to: Track outcomes after orthopaedic surgery; Link PROs to a systemwide value-driven outcomes program; Monitor and improve population health and enhance clinical datasets to facilitate research	The Recovery Tracker aims to enhance patient care by: Enabling real-time symptom tracking after major surgeries; Facilitating communication, reducing symptom burden, supporting early intervention	Implemented a PROM to measure heart failure-related health status to: Inform clinicians’ understanding of the patient’s health status and facilitate communication; Ensure patient’s perception of disease status is accurately captured to inform decision-making
2. Barriers & Facilitators	Administrative barriers: Lack of a governance structure and changes in governance; lack of implementation framework Facilitators: support from the health system leadership, the clinical and university medical group, and the physician practice group; hiring PROMs research experts; psychometrician to guide instrument selection and validation work	Barriers: patient reluctance to engage with daily surveys, technical challenges, and limited staff resources Facilitators: patient education, automated reminders and alerts, and integration with existing patient portals	Barriers: inconsistency of patient completion; incorporating review of PRO results into clinic workflow; IT challenges with using PROMs in multiple languages; heterogeneity of clinic population Facilitators: ease of interpreting results; similar to questions clinicians ask; ability to embed results in clinic documentation; patient comfort with pre-clinic PROs; clinician familiarity with PRO from clinical trials
3. Training & Engagement	Providers and staff at patient care sites trained on roles, patient engagement, IT troubleshooting, inventory tracking, and assessment completion; Engagement on provider-, administrator-, and system-levels, including marketing to patients about the program	Training includes patient education pre-surgery, at discharge, and follow-up calls; Engaging patients with clear instructions and reminders has proven effective, leading to high response rates	Clinician champion led implementation with support from a senior mentor and division leadership. Multiple meetings held with stakeholders including clinic staff, supervisors, clinicians, and IT. Clinician champion provided extensive training, resources, and summaries, checked in regularly at initial implementation
4. Identifying Patients	Patients identified by practice site using an EHR algorithm based on an orthopaedic visit with a surgeon, non-operative orthopaedic provider, physical therapist, or physician assistant-certified	Patients undergoing major urologic oncology surgeries (e.g., radical prostatectomy, nephrectomy, radical cystectomy, and retroperitoneal lymph node dissection for testicular cancer) are automatically assigned PROMs based on procedure coding, ensuring accurate patient identification	All patients in heart failure clinic and patients with heart failure seen in alternate general cardiology clinic; questionnaire only available in English
5. Outcomes & Measures	PROMIS and PROMIS Computer Adaptive Tests (including physical function) encouraged at initial implementation, along with comorbidity screener, and other legacy measures	Recovery Tracker items adapted from NCI PRO version of Common Terminology Criteria for Adverse Events for surgical patients, including pain, fatigue, nausea, wound condition, and other post-surgery symptoms	Selected KCCQ-12 because of relevance for guiding clinician understanding of patient disease and facilitating patient-clinician discussions in a heart failure clinic
6. Frequency & Timing	Timing varies from days, weeks, tri-monthly to yearly by sub-specialty group based on clinical visit cadence. Prompts sent 7- and 3- days pre-visit	Delivered daily for 10 days post-discharge; frequency designed to detect early symptoms and adjust patient management as needed	PRO is collected at each visit and prompted 3 days and 1 day prior to visit, or at check-in
7. Administering & Scoring	All administration is electronic and offered via email, text, or tablet collection in clinic; Scoring occurs automatically and data can be viewed by item or total score	Administered electronically via portal and automatically scored into different tiers of concerning symptoms (e.g., intermediate concern or urgent concern)	PRO collected via patient portal and can be completed online at home when prompted by EHR app or email or at check-in via text; Scored automatically
8. Workflow	Email/text invitation 7 and 3 days prior to visit; if PROM is incomplete patient receives tablet at front desk check-in; scores available in flowsheet and/or smart/dot phrases in EHR note for provider to see. In some cases, provider can drill down to item level responses	Incorporates regular monitoring of responses, with alerts reviewed by clinical team. Workflow adjustments were necessary to manage alerts efficiently, including automated notifications and staff coordination for follow-ups	Medical assistants document if patient completed PRO during check-in. Clinician workflows to access and use the data varies. Many clinicians have incorporated the data into their clinical notes, so they review PRO results while seeing the patient
9. Results Presentation	Provider dashboard has a report that graphs stratified scored assessment, and patient dashboard includes color/heat-map with percentile and age-adjusted score	Symptom data is presented to clinical teams in a structured format, allowing for quick identification of issues. Visual displays help patients and clinicians understand recovery status.	Numeric results available in tabular format and graphical format in EHR. Results can be automatically embedded in clinical note, tracked longitudinally, or viewed on a synopsis page with other clinical data
10. Visualizations	PRO visualization in EHR is a line graph	Uses color-coded alerts and clear text to indicate severity of symptoms and facilitate rapid interpretation and decision-making by both patients and healthcare providers	PRO visualization in EHR is a line graph
11.Responding to Issues	No alerts are sent to orthopaedic providers for concerning scores	Preset thresholds prompt response: “red alerts” instruct patients to seek immediate help, while “yellow alerts” prompt clinician follow-up during office hours	PRO is only collected at the time of visits, which was required during the initial build by the IT team to ensure an established interaction to address responses, as needed
12. Evaluation	There is currently no evaluation plan	Assess implementation outcomes (e.g., response rates, patient engagement) and clinical outcomes (e.g., reduced urgent care visits) to continuously refine system	Patient-level randomized trial showed that routine assessment improved patient experience and clinician assessment accuracy but did not impact clinical outcomes
13. EHR Integration	Integrated with EHR using PROMIS Application Programming Interface for scoring of PROMIS measures where available using EHR Flowsheets	Integrates data into EHR, allowing for seamless updates to patient charts and access to real-time information	Full EHR integration to ensure clinicians have data access as part of clinical workflow
14. Governance	Steering committee within orthoapedics that included the chair, project staff, and representative surgeons governed initial development and implementation	Involved setting policies for data handling, response protocols, and system updates. Multidisciplinary input ensures system aligns with clinical goals and ethical standards.	IT team governs PRO questionnaires in EHR system. Clinical division governs decision to support efforts from PRO licensing to requesting clinic staff support for PRO collection
15. Data Pooling & Exchanging	Data stored in electronic data warehouse; can be pulled directly through a data request form	Data stored in centralized database; analyzed to inform clinical guidelines and improve recovery care	Data stored in the clinical data warehouse and can be accessed for secondary research
16. Ethical & Legal Issues	Umbrella retrospective IRB (minimal risk) by broad orthopaedic conditions to access data with chair approval; IRB updated annually	Protocols ensure compliance with healthcare regulations and address liability concerns related to urgent alerts	Some concerns regarding lack of access to the instrument in other languages

*EHR* electronic health record, *IRB* institutional review board, *IT* information technology, *PRO* patient-reported outcome, *PROM* patient-reported outcome measure, KCCQ-12 Kansas City Cardiomyopathy Questionnaire

**Table 2b.** Case studies of PRO integration across multiple systems, summarized using the PROTEUS-Practice Framework

PROTEUS-Practice Framework step	Emma Children’s Hospital, Amsterdam UMC and their KLIK PROM portal	AmbuFlex–Center for Patient Reported Outcomes
1. Defining Goals	Pioneered the development and implementation of the KLIK PROM portal (www.hetklikt.nu) in 2011. The portal supports the use of PROMs in over 64 disease groups in 40 different hospitals to: Empower patients, enhance patient-clinician communication, and promote patient-centered care; Enhance research projects and care quality improvement using a dashboard with aggregated data	Public organization in Central Denmark Region that develops and supports the implementation of PROs across the Danish healthcare system. Over 80 disease-specific PRO solutions used across hospitals to: Enhance communication, identify issues, monitor treatment effects, support personalized clinical pathways/treatment; Deliver patient-centered care, improve care quality, and optimize resource use within the healthcare system
2. Barriers & Facilitators	Patient barriers: irrelevant PROMs and time Provider barriers: low-response rate, perceived time burden, irrelevant PROM content, technical aspects, and no full- integration with EHR Administrative barriers: involving and motivating stakeholders; tension between providing optimal support and cost of care Facilitators: identify and prepare champions; assess key stakeholders for readiness; inform local opinion leaders	Patient barriers: remote interaction feels impersonal, lack of confidence in one’s own ability to make decisions, and lack of understanding of PROM purpose Clinician barriers: mechanical and impersonal care, lack of confidence in the validity of PRO data, and increased workload Patient facilitator: better preparation and improved communication, improved insight into the disease, and more flexibility in daily life Clinician facilitator: better preparation, improved patient-centered focus, and efficient workflows
3. Training & Engagement	Training: Clinicians are trained in the theoretical components of PROM use in daily clinical care and practical aspects of the KLIK PROM portal Engagement: All stakeholders (patients, parents, clinicians) engaged; clinicians, PROM experts/psychometricians and sometimes patients and caregivers are involved in PRO and PROM selection; clinicians engaged in workflow decisions; patients increasingly involved in development of supportive materials like educational videos on PRO consultation	Training: PRO facilitator organizes clinician training sessions on integrating PROMs into practice, covering purpose, impact on workflows, communication strategies, and software functionalities Engagement: Active involvement of stakeholders is essential throughout development and implementation; managed by PRO facilitators through iterative meetings with a working group based on contextual requirements
4. Identifying Patients	Implemented from the bottom-up, with a request from care teams for PROM implementation in particular patient groups	PRO solutions generally implemented for a specific patient group (e.g., epilepsy, rheumatology, diabetes), most frequently in adults, but also for pediatric patients and their parents. Sometimes specific in- or exclusion criteria are used (e.g., linguistic skills, cognitive difficulties)
5. Outcomes & Measures	All stakeholders (patients, caregivers, clinicians) are ideally involved in selecting PROs; expert team advises on reliable, sensitive, and valid PROMs for selected PROs. Generic PROMs used when possible (preferably PROMIS); disease-specific PROMs used if necessary	PRO facilitator works closely with patients and clinicians to select clinically relevant PROMs following three steps: (1) Define content, (2) Pilot test in the target population, and (3) Define the PRO-based algorithm
6. Frequency & Timing	Varies for every multi-disciplinary team and patient group; typically used to monitor patient’s functioning over time, identify problems, provide tailored advice, and intervene early	Timing of when to complete PROMs is based on how PROMs are used in a specific PRO solution; frequency varies from daily, weekly, monthly, and yearly based on existing patient pathway
7. Administering & Scoring	Patients and/or caregivers complete PROMs online in portal at home (on average 2 weeks to 1 day) before the outpatient visit; The PROMs are scored automatically and converted into an electronic KLIK PROfile	Patients complete PROMs remotely online at home for the majority of the PROs, and scoring is automatic
8. Workflow	Different for every multidisciplinary team based on their needs and specific context. Generally, follows this workflow: invitation letters are sent to (new) patients; patients create portal account online and complete PROMs online at home; providers discuss at visit; automatic e-mails and reminders sent to patients when PROMs available for follow-up visit	Different for every PRO solution based on needs and specific clinical context. Consists of three general steps: 1. Enrolling patients in PRO solution 2. Collecting PRO responses from patients 3. Reviewing incoming PRO responses
9. Results Presentation	PROM results immediately available and visible to provider and patients and/or caregivers. Feedback options include literal representation of individual items, including traffic light color (red, yellow, green); summary scores; and graphical representations with norm lines	Results are presented to patients via a patient portal; PRO-based graphical overview tailored to each PRO solution is shown to clinicians within EHR
10. Visualizations	Has many feedback options that vary by PROM	Color-codes and bars are presented to clinicians. If a PROM score is used, detailed information about score interpretation is available. Clinicians are involved in the development of the graphical presentation of items and PROM scores to ensure that the visualization of PROMs aligns with their wishes and needs
11.Responding to Issues	No alerts are sent to clinicians about elevated scores. Dashboard shows literal and graphical representation of the completed PROMs. When cut-off scores are available for a specific PROM, these scores are helpful to the clinician. PRO scores are not intended to serve as stand-alone information, but rather as an integral component of clinical practice. It is therefore consistently emphasized that these scores should be discussed with the patient	If PROMs are used between or instead of clinical visits, alerts are sent to the clinicians. Red, yellow, and gray (non-responder) questionnaire responses are shown to the clinicians on an alert list. When PROs and algorithms are used for triage purposes, they are classified as medical devices under the European Union Medical Device Regulation and must comply with its guidelines
12. Evaluation	As a standard part of the implementation process, use of the portal is evaluated annually with each multidisciplinary team	PRO facilitator regularly monitors PRO usage, assessing whether an in-person visit is needed to address any issues, to ensure they meet user requirements, to support organizational efficiency, and to provide clear benefits for both patients and clinicians; A joint meeting with all relevant stakeholders is arranged as needed; A more in-depth evaluation of the PRO solutions takes place as needed
13. EHR Integration	Since September 2019, the portal has front-end integration with the two most often used EHRs in the Netherlands in four hospitals	Since March 2012, the system has been integrated via a link in the EHR in the Central Denmark Region. Since 2019, it has been integrated via a link in the EHR in North Denmark Region
14. Governance	Although a government-initiated program devoted significant attention to PROs and PROMs, there remains a proliferation of questionnaires and systems	AmbuFlex, Centre for Patient-reported Outcomes is a public organization located at Gødstrup Hospital in the Central Denmark Region. The region has a local PRO governance structure and is involved in a national PRO network
15. Data Pooling & Exchanging	Dashboards with aggregated data recently became available, where information is presented at the group level to enable comparison of responses between different patient groups or hospitals, facilitating the use of this data for quality improvement	PROM data from PRO systems are stored locally in a secure server; PROM data also transferred to a regional data warehouse, maximizing the utility of aggregated PRO data for monitoring and quality improvement
16. Ethical & Legal Issues	Portal’s main purpose is for use in daily clinical care, but data also used for research with appropriate disclosures and informed consent	The main purpose of the PRO system is use in daily clinical practice, but data is also used for research with relevant ethical and legal approval. PRO data are governed under the same rules as other EHR data

*EHR* electronic health record, *PRO* patient-reported outcome, *PROM* patient-reported outcome measure

## Case studies on integrating PROs within an individual health system (Table 2a)

### University of Utah Health System

The University of Utah implemented the mEVAL (My EVALuation) system to collect PROs in its orthopaedic clinics beginning in 2014, with expansion across the health system beginning in 2015. Initially focused on tracking surgical outcomes and supporting value-driven care, the system evolved to include a wide range of Patient-Reported Outcomes Measurement Information System (PROMIS) and other legacy measures across multiple clinical departments [22–25]. PROMs are administered electronically through mEVAL via email, text (where available), or iPad, with scoring for many measures provided, including PROMIS-specific measures integrated through the PROMIS Application Programming Interface [26, 27]. Implementation was initially supported by strong C-suite leadership, dedicated information technology (IT), clinical staff, and infrastructure investments. However, sustainability was challenged by leadership turnover, lack of a formal governance structure, and shifting departmental priorities. While workflows were initially well-supported, engagement waned over time, particularly during the COVID-19 pandemic, especially as iPADs were removed or re-deployed. Despite these challenges, the system continues to support research and clinical care, with data stored in an enterprise data warehouse and used for Human Subjects approved secondary data analysis, as well as accessible to clinicians via Epic flowsheets integration. Recently, there has been an interest in and a proposal to consolidate measures and migrate the mEVAL system to Epic-based questionnaires. The interest is in part driven by system-level quality reporting requirements (registries or designations) and payment requirements (e.g., Total Hip Arthroplasty/Total Knee Arthroplasty (THA/TKA) Patient-reported Outcome-based Performance measure (PRO-PM)). This proposal is currently being implemented by the health system and is guided by the PROTEUS-Practice Framework [17] and the ePROs Toolkit: Advancing Clinical Care through the Patient Voice [13]. 

## Memorial Sloan Kettering Cancer Center (MSKCC)–Recovery Tracker

MSKCC’s Recovery Tracker is a post-surgical symptom monitoring tool used for patients undergoing major urologic oncology procedures that was first launched in 2016. Patients complete daily symptom surveys for 10 days post-discharge, with automatic alerts triggered for concerning responses. The system supports early intervention, reduces symptom burden, and enhances communication. PROMs are administered via the patient portal, and results are integrated into the EHR for clinician review. Implementation included structured patient education and staff coordination, with high response rates achieved through reminders and clear instructions. Visualizations are designed for rapid interpretation, and alerts are tiered to guide clinical response. The system is continuously refined based on implementation and clinical outcomes data, and its integration into routine care has been supported by multidisciplinary governance and alignment with institutional goals.

## Stanford Health Care–Heart Failure Clinic

At Stanford’s heart failure clinic, the Kansas City Cardiomyopathy Questionnaire (KCCQ-12) [28] was implemented starting in 2021 to improve clinician understanding of patient-reported health status. Patients are prompted to complete the KCCQ-12 before each clinic visit via the patient portal, with results automatically scored and available in the EHR. The initiative was led by a clinician-investigator and supported by division leadership, with training provided to clinicians and staff. While the tool was well-received for its clinical relevance and ease of interpretation, barriers included inconsistent patient completion, workflow integration challenges, and limited language availability. The implementation was evaluated through a randomized trial, which demonstrated improved patient experience and clinician assessment accuracy, though not clinical outcomes [29]. Possible explanations for lack of impact on clinical outcomes include variable clinician adoption of the tool, limited incorporation of PRO data into routine clinical decision‑making, modest expected effect sizes, and relatively preserved baseline health status among trial participants. These findings suggest that access to PRO data alone may be insufficient to change outcomes without sustained clinician engagement and meaningful integration into routine-decision making. The system remains in use, with ongoing efforts to improve accessibility and clinician engagement.

### Comparison of case studies using PROs within an individual health system

These three case studies illustrate diverse approaches to PROM implementation, each tailored to specific health system contexts. The University of Utah’s orthopaedic program led to a system-wide integration that was supported by a robust technical infrastructure and provides research utility but was challenged by sustainability and governance gaps. It also highlights how interest in PRO integration can be driven by quality reporting requirements and performance measures. In contrast, MSKCC’s Recovery Tracker focused on short-term, high-frequency symptom monitoring with strong alignment with clinical workflows and real-time response protocols. Stanford’s heart failure clinic adopted a disease-specific PROM to enhance communication and decision-making, with a focus on embedding the tool into routine care and evaluating its impact through rigorous study.

While all three systems leverage EHR integration and electronic PROM administration, their approaches to frequency, workflow integration, and response mechanisms vary. Utah’s mEVAL system supports longitudinal tracking with variable timing, MSKCC's Recovery Tracker uses daily monitoring with automated alerts, and Stanford's heart failure clinic uses visit-based assessments with pre-visit prompts. Each case also highlights the importance of leadership support, stakeholder engagement and training, though the extent of formal governance and evaluation differs.

## Case studies that have integrated PROs across health systems (Table 2b)

### Emma Children’s Hospital, Amsterdam UMC – KLIK PROM Portal

Since 2011, Emma Children’s Hospital has supported large-scale integration of PROs across 64 disease groups and 40 hospitals in the Netherlands [30, 31]. Starting in 2025, they have also included some primary care settings, including in pediatric primary care physical therapy settings. This expansion represents the first use of KLIK outside hospital environments, and there are plans to extend the portal to adult primary physical therapy care. Patients—both pediatric and adult—complete PROMs remotely prior to outpatient visits, generating a personalized dashboard (KLIK ePROfile) that clinicians use to guide care discussions and interventions [32, 33]. Emma Children’s Hospital financially supported the initial development of the KLIK PROM portal and part of the initial implementation. Currently, it is a combination of financial support by the Emma Children’s Hospital and individual care teams pay licenses to use KLIK. Implementation is initiated by multidisciplinary teams for specific patient groups and stakeholder engagement is central to the process, with clinicians, PROM experts, patients, and caregivers [34] involved in selecting PROMs, shaping workflows, and developing educational materials. The basis for selecting PROMs is a generic set, adding more disease specific PROMs when necessary. Clinicians receive structured training, and the system is evaluated annually with the clinicians to identify barriers and improve functionality [35]. The portal supports both clinical care and research, with recent enhancements including aggregated dashboards for quality improvement. Emma Children’s Hospital emphasizes PROs as tools for patient empowerment, improved communication, and early detection of health concerns.

### AmbuFlex–Center for Patient-Reported Outcomes

AmbuFlex is a centralized, publicly funded initiative that has implemented over 80 disease-specific PRO solutions across hospital departments in two Danish Regions since 2011 [36, 37]. PROMs are completed remotely, scored automatically, and visualized using color-coded alerts embedded in the EHR. A practical, generic model is used for developing, implementing, and maintaining PRO solutions in clinical practice, but the implementation process is highly flexible and co-created. Each PRO solution is tailored to its clinical context, with structured processes for stakeholder engagement, PROM selection, and clinician training led by dedicated expert PRO facilitators. A three-step clinical workflow guides patient enrollment, data collection, and review of results. Visualizations are co-designed with clinicians and used to support clinical decision-making. Data are routinely transferred to a regional warehouse to support system-wide evaluation and improvement. AmbuFlex emphasizes using PROs to deliver patient-centered care, improve quality, and optimize resource use.

### Comparison of case studies that have integrated PROs across multiple health systems

Both the KLIK PROM portal and AmbuFlex demonstrate long-standing, multi-system integration of PROs to support patient-centered care and clinical decision-making. An underlying key difference is their funding structure, which influences multiple considerations across the PROTEUS-Practice Framework. The KLIK PROM Portal was initially supported by Emma Children’s Hospital, but its continued use is sustained through licenses paid by individual care teams as well as funding from the Emma Children’s Hospital. In contrast, AmbuFlex is publicly funded which enables more centralized and coordinated integration. This difference in funding is reflected in each group’s technical infrastructure. The KLIK PROM portal was developed through a long-standing collaboration with an external IT organization whereas AmbuFlex maintains an internal IT team.

Despite these structural differences, the two systems share several foundational features, as detailed in Table 2b. They both rely on remote completion of PROMs, automatic scoring of responses, and active stakeholder engagement throughout the integration process. Each system also uses a team-driven, flexible approach that allows for the co-creation of workflows, PROM collection frequency, and visualizations customized by multidisciplinary teams according to clinical needs and context.

However, differences emerge across several considerations of the PROTEUS-Practice Framework. One such area is how each system is designed to respond to potentially concerning PROM scores. The KLIK PROM portal does not send automated alerts; instead, PROMs are intended to be reviewed as part of the clinical conversation and are not used as stand-alone triggers for action. AmbuFlex takes a context-dependent approach. When the purpose of the PROM is to support discussion, clinicians review PROMS prior to and/or during a visit. However, AmbuFlex can also use a color-coded, alert-based triage approach when this aligns with the goals of the PRO solution. For example, it can use an alert-based triage approach to determine the need for an outpatient appointment, when PROMs are used between or instead of clinical visits. EHR integration also varies between the systems. While both have achieved some level of integration, the KLIK PROM portal offers front-end integration, and only in four hospitals. AmbuFlex, by comparison, has achieved broader regional linkage with all hospitals in two of Denmark’s five regions having access to the AmbuFlex system via the EHR. Finally, the systems differ in their approaches to data pooling and system-wide analysis. AmbuFlex routinely transfers PROM data to a regional warehouse for use on an aggregated level for monitoring purposes and quality improvements. The KLIK PROM portal, in contrast, has more recently introduced aggregated dashboards that support local quality improvement by enabling comparisons across patient groups and hospitals. These differences reflect how each system adapts PRO integration to its funding model, infrastructure, and national context while maintaining core principles of patient-centered care and clinical utility.

### Cross-cutting lessons learned

Fifteen cross-cutting lessons emerged across the three stages of the PROTEUS-Practice Framework, along with additional unique lessons learned for some case studies (Table 3).

**Table 3:** Key lessons learned from case studies

PROTEUS-Practice Framework stage	Cross-cutting lessons learned	Unique lessons learned
Design	Understand the rationale behind using PROMs and define relevant clear goals to shape the PRO system; Engage all relevant stakeholders (clinicians, patients, PROM experts, leadership); Train all stakeholders, including clinicians and patients, on value and use of PRO systems; Assess organizational readiness to help prevent and/or address potential barriers; Align expected outcomes with the overall purpose of using the PRO system in a specific clinical context; Implement from bottom-up implementation to promote success; Use flexible, co-creation approach with PRO and clinical experts to customize PRO integration	*University of Utah Health System*: Staff turnover requires regular training; *Stanford’s Heart Failure Clinic*: Use multiple approaches for data collection
Implementation	Identify champions on the healthcare team who can support training and implementation, help overcome barriers, and serve as a role model for use; Provide initial and ongoing training and engagement at all health system levels, particularly among clinicians and patients; Offer easy access to and clear visualization of PROM results for clinicians and patients; Use iterative development and integration approach with IT support to align the system with clinical workflows and user needs and wants	*KLIK PROM Portal*: Financial support initially received from healthcare system leadership with continued use sustained through licenses paid by individual care teams; *University of Utah Health System*: Important to give researchers an administrative or operations title so that staff see that this is not just another research project; *Stanford’s Heart Failure Clinic*: Multiple data collection approaches emphasized because the easiest time to collect the data - during visits- may not be when it is most valuable
System and Data Management	Integrate with EHR to promote PRO system feasibility and ease of use, despite the challenges involved and effort required; Conduct regular system updates and provide ongoing support to ensure functionality; Adhere to regulatory guidelines and data privacy laws, although doing so can impact PRO system usability; Implement a robust data management plan that prioritizes secure data handling and ongoing, regular system updates	*KLIK PROM Portal*: Full EHR integration and data privacy legislation are significant factors; *AmbuFlex*: Ongoing maintenance and adherence to regulations are crucial; *University of Utah Health System*: Strong system level leadership in both quality and IT with financial support needed to support EHR integration and sustainability

*EHR* electronic health record

*IT* information technology

*PRO* patient-reported outcome

*PROM* patient-reported outcome measure

Seven cross-cutting lessons were identified for the design stage. All case studies emphasized training for all stakeholders–clinicians, patients, PRO experts, and health system leaders–as critical for successful integration because it promotes trust, a sense of value, and uptake. Other common lessons learned included clearly defining goals for PRO use, engaging a broad range of stakeholders, assessing organizational readiness, and aligning expected outcomes with the system’s purpose. Long-standing initiatives, such as the KLIK PROM portal and AmbuFlex, demonstrate that early and broad stakeholder engagement helps tailor systems to local needs and fosters a sense of ownership. Bottom-up implementation and co-creation with clinical and PRO experts were also noted as valuable. Unique lessons learned included designing for flexible data collection approaches (Stanford Heart Failure Clinic) and planning for staff turnover through regular training opportunities.

Four cross-cutting lessons emerged for the implementation stage, where success was closely tied to the presence of local champions, provision of ongoing training, accessible and user-friendly visualizations, and iterative development to fit into clinical workflows. Champions play a critical role in training, troubleshooting, and modeling the use of PROs. Iterative design processes allow systems to evolve in response to user feedback, especially when there is IT team support. Visual clarity and ease of access to PROM results are essential for clinician and patient engagement. For example, MSKCC’s Recovery Tracker uses tiered alerts and structured workflows to support real-time symptom monitoring, while Stanford’s Heart Failure Clinic embeds PRO results directly into clinical notes. Unique lessons included challenges when PROs are perceived as research tools rather than clinical assets and the need to secure funding beyond initial financial support for launch.

Four cross-cutting lessons were identified for the management stage. These lessons emphasized the importance of robust infrastructure, governance, and compliance with data privacy regulations. EHR integration was widely recognized as essential for usability and feasibility, though it was noted that it often requires significant time, monetary, and technical resources. Regular system updates and ongoing support are necessary to maintain functionality and user engagement. Governance structures vary, from centralized oversight (AmbuFlex) to department- and physician-led steering committees (University of Utah), but all emphasize the need for multidisciplinary input. Data pooling and exchange are facilitated through regional warehouses or dashboards, enabling quality improvement and research. Unique lessons included AmbuFlex’s experience with European Union Medical Device Regulation compliance and the need for strong leadership and financial support for quality and EHR integration at the University of Utah.

## Discussion

By applying the PROTEUS-Practice Framework, we systematically described a diverse range of real-world case studies for integrating PROs in clinical practice across design, implementation, and management stages. Collectively, the five case studies demonstrate the importance of aligning PRO integration with different clinical needs while accounting for national context and organizational goals, infrastructure, and funding. Our findings reinforce and extend upon the implementation science literature by demonstrating the importance of tailoring strategies to local contexts [8]. Case studies such as AmbuFlex’s and the KLIK PROM portal’s flexible, team-driven approach illustrate how governance structures and stakeholder engagement help adapt to institutional culture and resources. These examples also reflect widely recognized implementation principles, including innovation compatibility, access to knowledge, and proactive planning [10]. For instance, MSKCC’s Recovery Tracker shows how structured workflows and tiered alerts can enhance alignment with clinical routines, while Stanford’s use of pre-visit prompts supports effective planning and patient engagement.

We also found strong support for calls to prioritize inclusive design and multimodal data collection strategies to ensure that PROM systems are accessible to all patients, regardless of language, literacy, or technological familiarity [38]. The need is reinforced by research conducted by AmbuFlex showing socioeconomic disparities in PROM completion rates [39, 40]. Stanford’s heart failure clinic exemplifies inclusive design by offering multiple options for PROM completion, including pre-visit prompts via the patient portal and in-clinic check-in via text, which helped accommodate varying levels of digital access and comfort. Despite barriers such as limited language availability, this flexibility in data collection modalities reflects a commitment to reaching all patient populations. More broadly, our findings highlight the value of digital health tools, such as electronic PROMs, in promoting broader access and enabling patients to use assistive technologies available through institutions or already available at home (e.g., adapted speech devices, or large font displays) [4]. 

Although this was a retrospective application of the PROTEUS-Practice Framework, the findings highlight areas where prospective use might have mitigated issues. Guidance on many considerations now emphasized in the Framework—such as PROM selection, stakeholder engagement, questionnaire burden, and visualization—were either not available at the initial time of PRO integration for some case studies or were addressed in more focused recommendations, but not in a unified, comprehensive manner. Additionally, while no case study reported using a single implementation science framework, many drew on established frameworks or implementation strategies over time as they started to be more actively applied in clinical practice [41–45], reinforcing the complementary role of PRO‑specific and general implementation approaches. The PROTEUS-Practice Framework brings these considerations together in a PRO‑specific, operational structure that may support more efficient and scalable implementation. For example, several challenges encountered by case studies align with considerations in the Framework and may have benefited from earlier or more deliberate attention, including staff turnover, inconsistent training, or delayed establishment of governance structures. Several contributors noted that earlier access to a consolidated, PRO‑specific framework may have reduced iterative redesign and variability across clinics or institutions.

Nevertheless, this study has several limitations that should be considered when interpreting its findings. First, the case studies represent a convenience sample drawn from participants in the PROTEUS Consortium. The selection process was not designed to be comprehensive or representative of global PRO integration efforts, and the included examples varied in maturity, scope, and depth across the 16 framework steps. As such, generalizability may be limited. Second, while the structured template based on the PROTEUS-Practice Framework provided consistency across submissions, our analysis relied on summative thematic synthesis rather than other qualitative methods such as coding, triangulation, or member checking. This approach may have missed nuanced differences across contexts or underrepresented divergent experiences. Finally, while the PROTEUS-Practice Framework offers a comprehensive structure, it was applied retrospectively and descriptively rather than evaluatively; we did not assess the relative effectiveness of different approaches or outcomes across case studies.

This work makes a novel contribution by offering practice‑based, PRO‑specific operational insights derived from real‑world PRO integration and systematically organized using the PROTEUS‑Practice Framework. Rather than focusing on high‑level barriers and facilitators alone, this study explicitly describes the concrete design, implementation, and management decisions that shaped how PRO systems were used in varied care settings. These findings complement general implementation science approaches and highlight opportunities for future research, including prospective application of the PROTEUS‑Practice Framework, assessment of its impact on implementation outcomes (such as PRO completion rates, usability, and care processes), and testing of its applicability across additional healthcare settings and contexts, including resource‑constrained environments.

Successful PROM interventions require more than technical integration, they demand alignment with clinical goals, patient needs, and organizational capacity. The PROTEUS-Practice Framework offers a structured yet flexible roadmap for navigating these complexities. The case studies highlight the importance of addressing all 16 steps of the Framework. By synthesizing lessons learned from real-world case studies, this work contributes actionable guidance for optimizing PRO integration into clinical care in ways that are sustainable, scalable, and inclusive.

## Data Availability

All data supporting the findings of this study are available within the paper.
